# Treatment According to Molecular Profiling in Relapsed/Refractory Cancer Patients: A Review Focusing on Latest Profiling Studies

**DOI:** 10.1016/j.csbj.2019.03.012

**Published:** 2019-03-26

**Authors:** Kai Zimmer, Florian Kocher, Gilbert Spizzo, Mohamed Salem, Guenther Gastl, Andreas Seeber

**Affiliations:** a Department of Haematology and Oncology, Medical University of Innsbruck, Austria; b Levine Cancer Institute, Carolinas HealthCare System, Charlotte, USA

**Keywords:** Molecular profiling, Personalized medicine, Precision oncology, Metastatic cancer, Abl, Abelson murine leukemia viral oncogene homolog 1, ASCO, American Society of Clinical Oncology, Bcr, Breakpoint cluster region, CGH, Comparative genomic hybridization, CISH, Chromogenic in-situ hybridization, CR, Complete response, DNA, Deoxyribonucleic acid, FDA, Food and Drug Administration, FGFR, Fibroblast growth factor receptor, FISH, Fluorescence in-situ hybridization, HER2, Human epidermal growth factor receptor 2, HR, Hazard Ratio, IHC, Immunohistochemistry, MEK, Mitogen-activated protein kinase, MP, Molecular profile, MSI, Microsatellite Instability, mTOR, Mammalian target of Rapamycin, NCI, National Cancer Institute, NGS, Next generation sequencing, ORR, Overall response rate, OS, Overall Survival, PCR, Polymerase chain reaction, PFS, Progression-free survival, PIK3CA, Phosphatidylinositol-4,5-bisphosphate-3-kinase catalytic subunit alpha, PR, Partial Response, PTEN, Phosphatase and tensin homolog, RAF, Rapidly growing fibrosarcoma - protein, RNA, Ribonucleic acid, R/R, Refractory/Relapsed, SD, Stable Disease, TTF, Time to treatment failure, WES, Whole-exome sequencing

## Abstract

In this review we aim to summarize studies investigating the impact of a molecular profiling (MP)-guided treatment approach in heavily pretreated cancer patients. In summary, many independent single- and multicenter studies showed a significant benefit of MP-guided treatment regarding response rates and survival. However, in the only randomized trial conducted so far, no benefit of MP-guided targeted therapy was observed. Notably, various profiling approaches were conducted in the respective studies: some studies used a single analytic approach (i.e. next-generation sequencing), others applied multiple analytic methods to perform comprehensive molecular profiling. It seems that multiplatform profiling analyses, detected an increased number of druggable molecular targets or signaling pathway alterations and that a higher proportion of patients was treated according to the molecular cancer profile. Even though no randomized study has shown a benefit of molecular profiling so far, many studies indicate that MP-guided treatment can be beneficial in patients with relapsed and/or refractory cancer. Currently ongoing large randomized trials (i.e. NCI-MATCH, TAPUR) will add evidence to the role of profiling-guided cancer treatment.

## 1 Introduction

During the last twenty years the establishment of new analytic approaches and the exploration of the genomic landscape in the majority of cancer types has paved the way towards precision medicine in oncology. The aspiration of precision oncology is to change our clinical routine and to treat cancer patients according to their individual molecular profile. Especially, the development of rapid sequencing techniques, such as next-generation sequencing (NGS), resulted in a deeper understanding of the complexity of cancerogenesis and the heterogeneity of genetic alterations in malignancy. Many potential targets on genetic level, which are essential for proliferation, survival and metastatic spread of cancer cells, were identified. Some of those - so-called driver-mutations – have been elucidated as highly efficient therapeutic targets and nowadays there is a rapid progress in the development of new and more potent molecular targeted therapies to prevent or circumvent primary and secondary resistance mechanisms [1,2]. A prime example was the discovery of the Philadelphia chromosome and the underlying reciprocal Bcr/Abl translocation as the pathogenic event responsible for the development of chronic myeloid leukemia. The subsequent approval of imatinib, an inhibitor of this activated BCR/ABL-kinase, yielded impressive outcome data with the chance of cure after stopping treatment in patients with prolonged deep molecular remission [3]. In breast cancer patients, the overexpression of HER2 was linked to a poor prognosis [4]. Since the approval of trastuzumab, an anti-HER2 antibody, the outcome in this subset of patients has improved substantially [5]. Of note, several driver genes and related protein alterations such as HER2 are not limited to a certain tumor entity. Efficacy of HER2-inhibition has also been observed in gastric and colorectal cancer [6,7].

With gaining knowledge in cancer genomics, molecular biology and the development of targeted therapies, the classic concept of phase I-III trials has been extended by new clinical trial designs. Basket- and umbrella trials entered the field of clinical research. In basket trials, different tumor entities with a certain gene alteration are included for matched therapy, whereas in umbrella trials the mutational profile leads to the stratification to different targeted treatment approaches in one tumor entity. Promising early data has been generated in such trials. However, the importance of precision medicine in this setting remains controversial and for patients with relapsed and refractory (R/R) cancer the benefit of molecular profiling (MP)-guided treatment has still to be proven.

In this review, we aim to provide an update of published and ongoing studies using various profiling techniques in the field of precision cancer medicine in patients with R/R disease.

## 2 The Beginning

One of the first studies ever published in the field of precision medicine in R/R cancer patients, was a pilot-study conducted by D. Von Hoff in 2010. In this trial, MP was carried out using immunohistochemistry (IHC), fluorescence in-situ hybridization (FISH) and oligonucleotide microarray gene expression assay. One-hundred-and-six patients were enrolled and tumor tissue of 86 patients (81.1%) was finally analyzed. In 98% a molecular target was identified and in 66 patients (76.6%) a treatment according to the MP was conducted. In such trials progression-free survival (PFS) as an endpoint does not reflect efficacy, due to the entity-specific tumor heterogeneity. To evaluate the effectiveness of treatment and to identify those patients benefitting from the therapy in such a heterogeneous group, the PFS-ratio (= PFS after guided-treatment / PFS before guided-treatment) was defined as primary objective. A PFS ratio of ≥1.3 was arbitrarily considered as therapeutic efficacy, which means that PFS of guided treatment is 30% improved compared to the PFS achieved in the previous therapy line. This mode of efficacy evaluation has been used frequently in subsequent trials evaluating profiling-guided treatment. This threshold was exceeded by 18 patients (27%; *p* = .007) with a median PFS-ratio of 2.9. An overall response rate (ORR) of 10% was achieved in patients receiving MP-guided treatment [8].

In a non-randomized phase I trial, conducted by the MD Anderson Cancer Center initiative, 1144 patients with advanced cancer were profiled by polymerase chain reaction (PCR)-based sequencing and FISH. At least one aberration was detected in 40.2% (*n* = 460) of patients. In total, 22.4% (*n* = 256) were treated with a matched drug whereas 12.3% (*n* = 141) were treated with a non-matched drug, which served as a control group. An outcome analysis was performed for patients with only one aberration (68.3%, *n* = 175 matched therapy vs *n* = 116 in the control group), and for those who had 2 or 3 molecular alterations (23.8%, *n* = 61 matched therapy vs *n* = 25 in the control group). In patients with only one aberration detected, the ORR was 27% for matched therapy vs 5% in the control group (*p* < .0001). Stable disease (SD) ≥6 months was achieved in 23% in the matched group vs 10% in the control group. Median time-to-treatment failure (TTF) and median OS were significantly increased in those patients treated according to their MP (median TTF: 5.2 vs 2.2 months; *p* < .001; median OS: 13.4 vs 9.0 months; *p* = .017). Patients with 2 or 3 molecular alterations showed no increase in median TTF (3.0 vs 2.7 months; *p* = .79) and median OS (10.6 vs 17.0 months; *p* = .28). Overall, targeted therapy was identified as the major independent factor predicting higher rates of response (*p* = .001) and TTF (*p* < .001). Furthermore, a tendency towards longer survival (*p* = .06) was observed [9].

In a subsequent follow-up study incorporating 1276 tissue samples comparable results were observed [10]. Again, a multivariate analysis identified matched therapy as one independent factor predicting response (*p* = .015) and longer PFS (*p* = .004). Of note, in this analysis OS was significantly improved in the matched therapy cohort compared to the unmatched collective (11.4 vs 8.6 months; *p* = .04).

## 3 Single Center Experiences

The PREDICT-trial of the UC San Diego Moores Cancer Center profiled 347 patients with solid advanced cancers by NGS, and a total of 25% (*n* = 87) were treated according to their genomic profile. A PFS-ratio ≥ 1.3 was reached in 45.3% of patients treated with a matched therapy vs 19.3% in the control group (*p* = .004). No difference in OS was observed. Interestingly, the group developed a matching score that divided the number of matching drugs by the number of aberrations. Those patients with a matching score > 0.2 had a higher median OS compared to patients who had a score < 0.2 (15.7 vs 10.6 months; *p* = .04). Matched therapy remained the only significant independent factor associated with a SD ≥ 6 months (*p* = .02) [11].

Wheler et al. conducted a single center study including 500 patients in various refractory solid tumor types. Of those, 339 (67.8%) were profiled by NGS performed by a commercially-available profiling service. A potentially actionable target was detected in 93.5% (*n* = 317). Finally, treatment was initiated in 37.6% (*n* = 188) of all enrolled patients, 68% (*n* = 122) with a matched and 32% (*n* = 66) with an unmatched therapy. Fewer previous therapy lines were associated higher rates of SD ≥ 6 months. When a matching score comparable to the one used in the PREDICT-trial was applied, it revealed that high matching scores were independently associated with a greater frequency of SD ≥ 6 months (22% with a high vs 9% with a low matching score; *p* = .024), longer TTF (HR = 0.52; *p* = .0003) and survival (HR = 0.65; *p* = .05) [12].

The Princess Margaret Cancer Center published the results deriving from their IMPACT and COMPACT trials, in which 1893 patients with different advanced solid tumors were enrolled. In 1640 (87%) patients genomic sequencing was performed. Genetic aberrations were identified in 341 patients (41%), but only a minority (*n* = 84, 5%) was treated according to the detected aberrations. ORR for those patients receiving anticancer therapy was higher in the matched, compared to the non-matched treatment cohort (19% vs 9%; *p* = .026). Multivariate analysis revealed ORR superiority in women and matched therapy according to the cancer genotype [13].

Updated results of the IMPACT trial were presented at the annual meeting of the American Society of Clinical Oncology (ASCO) in Chicago in 2018. Between 2007 and 2013, 3743 patients underwent MP, of which 34.9% had at least one targetable molecular alteration. A comparison of the outcome in patients treated with a targeted approach (54.4%, *n* = 711) and patients treated with a non-matched therapy (45.6%, *n* = 596) revealed a significantly higher response rate in the matched therapy group (34.9% vs 20.1%, *p* < .001). As this trial followed patients for over 10 years, long-time outcome was presented. OS rates were 15% vs 7% after three years and 6% vs 1% after 10 years (*p* < .001), favoring the matched therapy approach [14].

The SAFIR01/UNICANCER-trial enrolled 423 patients with metastatic breast cancer. Two-hundred-ninety-nine (70%) patients were profiled by comparative genomic hybridization (CGH) and Sanger sequencing with regard to PIK3CA and AKT1 aberrations. In 195 (46%) patients, at least one actionable alteration was detected and 55 patients (13%) received matched treatment. Of the 43 evaluable patients, a disease control rate above 16 weeks was achieved in 30% (*n* = 13) [15].

Kris et al. conducted a prospective study, in which 1007 patients with metastatic lung-cancer were analyzed for 10 specific oncogenic driver mutations. In 64% (*n* = 466) of 733 patients an oncogenic driver was found. Finally, targeted therapy was conducted in 28% of patients. Median OS was significantly improved in the patients with an oncogenic driver and genotype-directed therapy (*n* = 260), in comparison to the patients without any oncogenic driver who did not receive genotype-directed therapy (*n* = 318) (3.5 vs 2.4 years; *p* = .006) [16].

## 4 Multiplatform Profiling

Most studies presented in the previous sections focused on DNA sequencing. However, to detect more actionable targets, a combination of DNA sequencing with other techniques, such as RNA-sequencing or IHC may provide additional suitable targets. As such, the studies presented below focused on multiplatform profiling.

In the recently published MOSCATO-01 trial, 843 patients with refractory solid cancers underwent a multiplatform profiling (array-based CGH, RNA-sequencing, whole exome sequencing [WES]). In 411 (49%) patients, a potentially actionable target was detected and a total of 199 patients (24%) received a matched therapy. The PFS-ratio exceeded 1.3 in 33% of those patients (*p* < .001), ORR was 11% and the median OS was 11.9 months [17].

In 348 patients with ovarian cancer, multiplatform MP was performed by a commercially available profiling center and included Sanger sequencing, NGS, pyrosequencing, IHC, FISH, chromogenic in-situ hybridization (CISH) and RNA-fragment analysis. In total, 170 (48.8%) patients were assigned to a group treated with profile-guided targeted agents and were compared to 178 (51.2%) patients with unmatched therapy. MP-guided treatment lead to a significantly longer post-profiling survival (HR 0.54; *p* = .0018) [18].

At the annual meeting of ASCO in 2017 an interim-analysis of the still ongoing PROFILER trial (NCT01774409) was presented. In this trial multiplatform profiling was conducted in 2490 patients with solid cancers. In 51.5% (*n* = 940) at least one actionable mutation was found using exome sequencing and CGH. Treatment options were discussed in a molecular tumor board and resulted in a MP-guided targeted therapy in 101 patients (10.7%). Median PFS was 2.8 months, 2 patients (2.3%) had CR, 13 patients (15.1%) had PR and 29 patients (33.7%) had SD [19]. Final results of the trial are expected in 2020.

Investigators from the University of Michigan enrolled 102 adolescent patients (median age 11.5 years) with refractory and relapsed malignancies, and performed a MP using exome and transcriptome sequencing. They identified potentially actionable targets in 54% (*n* = 15) of patients with hematological malignancies and in 43% (*n* = 27) of patients with a solid tumor. This resulted in a change of treatment in 14 patients leading to an ongoing PR (lasting 8–16 months) or CR (lasting 6–21 months) in 9 patients (64%) [20].

Bryce et al. analyzed 141 tumor specimens of patients with hematological and solid malignancies. Genetic testing included NGS, CGH and WES. Results were classified as “actionable”, in cases of specific mutations with the option of treatment with FDA-approved targeted therapies, or as “informative”, defined as a prognostic marker. In 65% (*n* = 92) an actionable mutation was identified, and informative mutations were detected in 73% (*n* = 103). Targeted therapy, as a result of genomic testing was provided in 31% (*n* = 29), of which 45% (*n* = 13) showed a clinical response [21].

Results of an early interim-analysis of the ONCO-T-Profile program were reported previously. Within this clinical program 110 patients with R/R metastatic tumors who had failed standard treatment were treated according to MP. MP included NGS, RNA-sequencing, IHC and FISH/CISH). At the time of analysis, 50 patients have been profiled and in 98% one or more druggable targets were detected. Of 19 patients treated according to MP a PFS-ratio ≥ 1.3 was achieved in 42% (*n* = 8) [22].

Results of the WINTHER - trial have been presented at the ASCO meeting in Chicago in 2018. Patients were either profiled by DNA-sequencing (NGS; Arm A) or RNA-profiling (transcriptomics performed by oligo-arrays; Arm B). One-hundred-and-seven patients were treated according to their MP (DNA-guided: *n* = 69 [64.5%]; RNA-guided: *n* = 38 [35.5%]), resulting in a SD/PR/CR > 6 months in 26.2% of patients (DNA-guided: 23.2%; RNA-guided: 31.6%). Median PFS was 2.1 months (DNA-guided: 1.9 months; RNA-guided: 2.4 months). A higher matching score was significantly associated with better PFS (*p* = .005) and OS (*p* = .012). However, the trial did not meet its primary endpoint of a PFS-ratio > 1.5 in 50% of patients in Arm A and a PFS-ratio > 1.5 in 40% of patients in Arm B. A full publication of the results is still awaited [23]. Several limitations had been identified by the primary investigators ahead of the trial, such as the study design (triage trial), the ambitious endpoint (PFS-ratio > 1.5 instead of > 1.3), the limitations of drugs used and the financial resources [24]. The upcoming SPRING and MERCURY trial by the WIN-Consortium aim to implement the lessons learned from the WINTHER - trial.

The interventional phase II, open-label, non-randomized, multicenter NCI-MATCH trial currently comprises 39 treatment arms with >6450 patients included. It is designed as a basket trial, treating patients irrespective of tumor histology. Molecular testing is performed by AmpliSeq (143 genes) and IHC. The primary objective is to evaluate the ORR in patients with refractory tumors. First results have been presented for 6 treatment arms at ASCO and ESMO meetings in 2018. In 37 heavily-pretreated patients with an HER2 amplification the use of trastuzumab-emtansin led to a SD in 43% and a 6 months PFS rate of 24.8% [25]. Fifty patients with aberrations in the fibroblast growth factor receptor (FGFR) were treated with the selective inhibitor AZD4547. ORR and SD rate were 5% and 51%, respectively. Fifteen percent achieved a duration of response longer than 24 weeks and the 6 months PFS rate was 17% [26]. Patients with PIK3CA-mutated tumors received taselisib, an oral specific PIK3CA inhibitor. The use of taselisib did not result in any objective response but the authors reported a 6 months PFS rate of 27% [27].

In two arms of the study patients with PTEN aberrations were treated with GSK2636771. In PTEN mut/del tumors (*n* = 22) PR and SD in 4.5% and 32% were observed. In patients (*n* = 34) with loss of PTEN (assessed by IHC) SD was achieved in 37.5%. Median PFS was 1.8 months for both arms [28]. Thirty-five patients harbouring AKT mutations were treated with capivasertib, yielding a PR in 8 patients (23%) and a SD in 16 patients (46%) [29]. Further results of the trial will be presented in the near future.

Main results of the presented studies and further major results of multiple single center analyses are displayed in Table 1.

**Table 1:** Overview of studies focusing on molecular profiling

Profiling mode	Author/Trial	Study design	Cancer type	Methods/FDA approved only	Patients enrolled - n	Patients profiled – n (% of enrolled)	Patients treated – n (% of enrolled)	Median survival (months) matched vs. unmatched	PFS-ratio > 1.3	Response (in %)
Next generation sequencing	Schwaederle et al. (PREDICT) 2016 [11]	Retrospective	solid tumors	NGS/No	347	347 (100%)	87 (25%)	PFS: 4.0 vs 3.0 *p* = .056; OS: 12.4 vs 14.4 *p* = .414	45.3 vs 19.3 p = .004	SD > 6 months/PR/CR: 34.5 vs. 16.1
	Wheler et al. 2016 [12]	prospective Matched vs. unmatched	solid tumors	NGS/No	500	339 (68%)	188 (55%)	TTF 2.8 vs 1.9, p = .001; OS: 9.3 vs 7.2 *p* = .087		SD > 6 months/PR/CR 19 vs 5 *p* = .061
	Stockley et al. (IMPACT/COMPACT) 2016 [13]	prospective	solid tumors	NGS/No	1893	1640 (87%)	84 (5%)	OS: 16 vs 13 *p* = .10	.	ORR: 19 vs 9 p = .026
Multiplatform profiling	von Hoff et al. 2010 [8]	prospective	solid tumors	IHC, FISH, ONMGEA/Yes	106	86 (81.1%)	66 (76.6%)		27	10
	Tsimberidou et al. 2012 [9]	prospective	solid tumors	PCR, FISH/No	1238	1144 (92%)	256 (22.4%)	TTF: 5.2 vs 2.2 p < .0001; OS: 13.4 vs. 9.0 p = .017		ORR: 27 vs 5 *p* < .0001, SD > 6 months: 23 vs. 10
	Tsimberidou et al. 2014 [10]	prospective	solid tumors	PCR, FISH/No	1276	1276 (100%)	143 (11.2%)	PFS: 3.9 vs 2.2 p = .001; OS: 11.4 vs. 8.6 *p* = .04		ORR: 12 vs 5 p < .0001, SD > 6 months 16.4% vs 12.3
	André et al. (SAFIR01/UNICANCER) 2014 [15]	prospective	breast cancer	CGH, Sanger Sequencing/No	423	299 (70.6%)	55 (18%)			ORR: 9, SD > 16 weeks: 21
	Mody et al. 2015 [20]	retrospective	solid and hematological cancer in children and adolescents	exome and transcriptome sequencing/No	102	102 (100%)	14 (13%)		n.a.	ORR: 10
	Massard et al. (MOSCATO-01) 2017 [17]	prospective	solid tumors	targeted sequencing, aCGH, RNA-seq, WGS/No	1035	843 (81%)	199 (24%)	OS: 11.9	33%	ORR: 11, SD: 52
	Bryce et al. 2017 [21]	prospective	hematologic and solid tumors	NGS, CGH, WES/N0	165	141 (85%)	29 (25%)		n.a.	ORR: 8
	Tredan et al. (PROFILER) 2017 [19]	prospective	solid tumors	Targeted exon sequencing, CGH/No	2490	1826 (73.3%)	101 (4%)	PFS: 2.8	n.a.	ORR: 17.4, DCR: 51.1
	Seeber et al. 2017 [34]	pooled-analysis	solid tumors	NGS, IHC, FISH/CISH/ all approved but off-label	202	202 (100%)	166 (82%)	PFS: 4.0	52%	n.a.
	Rodon et al. (WINTHER) 2018 [23]	prospective	solid tumors	NGS, Oligo-arrays/No	303	303 (100%)	107 (35%)	PFS: 2.1	n.a	SD >6 months/PR/CR: 26.2
Prospective randomized	Le Tourneau et al. (SHIVA) 2015 [30]	prospective-randomized	solid tumors	NGS, copy number alterations, IHC/all approved but off-label	741	496 (67%)	99 (20%) experimental, 92 control	PFS: 2.3 vs 2.0 p = .41	n.a.	n.a.
	Belin et al. (SHIVA post-hoc) 2017 [31]	post-hoc	solid tumors	NGS, copy number alterations, IHC	741	496 (67%)	99 (20%) experimental, 92 control		37	n.a.
Meta-analysis	Schwaederle et al. 2015 [32]	meta-analysis (phase I)	hematologic and solid tumors		32,149		8078 (25.1%)	PFS: 5.9 vs. 2.7 p < .001; OS: 13.7 vs. 8.9 p < .001		SD: 29.2 vs 6.2 p < .001
	Schwaederle et al. 2016 [33]	meta-analysis (phase II)	hematologic and solid tumors		13,203		2655 (24.4%)	PFS: 5.7 vs. 2.95 p < .001		SD: 30.6 vs 4.9 p < .001

Abbreviations: PFS = progression-free survival, OS = overall survival, ORR = overall response rate, RR = response rate, NGS = next generation sequencing, SD = stable disease, PR = partial response, CR = complete response, TTF = time to treatment failure, n.a. = not available, IHC = immunohistochemistry, FISH = fluorescence in-situ hybridization, ONMGEA = oligonucleotide microarray gene expression assays, PCR = polymerase chain reaction, CGH = comparative genomic hybridization, RT-PCR = real time polymerase chain reaction, CISH = chromogenic in-situ hybridization, WGS = whole genome sequencing, WES = whole exome sequencing, at.

## 5 Randomized Trial

The SHIVA-trial was a controlled phase II trial in the field of precision oncology using comprehensive molecular tumor profiling. Eight French medical centers enrolled 741 patients with solid tumors, of which 496 (67%) were molecularly profiled by NGS, copy number alterations and IHC. Patients were stratified according to different molecular alterations (e.g. hormone receptor-, PI3K/AKT/mTor- and RAS/RAF/MEK- pathway), cross over was permitted in case of progression. In total, 195 patients (26%) were randomly assigned to a treatment arm (99 experimental, 96 controls). PFS at 6 months was 13% in the control group vs 11% in the experimental group. The median PFS was 2.3 months in the experimental group compared to 2.0 months in the control group (HR 0.88, 95% CI 0.65–1.19, *p* = .41). The study failed achieve the pre-specified primary endpoint of 15–30% PFS improvement [30]. Based on the results the authors concluded, that off label use of molecularly targeted agents outside of clinical trials should be discouraged. In a post-hoc analysis of the SHIVA-trial, Belin et al. assessed the PFS-ratio of patients that crossed-over in the trial (*n* = 95). PFS-ratio exceeded 1.3 in 37% of the patients that crossed-over from the “Treatment at Physician's Choice” (TPC) to the “Molecularly Targeted Agent”-arm (MTA), whereas the PFS ratio exceeded 1.3 in 61% of the patients crossing-over from the MTA to the TPC arm [31].

Main targets and the respective agents used in the different trials are listed in Supplementary Table.

## 6 Meta- and Pooled–Analyses

Schwaederle et al. performed a meta-analysis of published data from phase II clinical trials in the setting of MP. A total of 32,149 patients recruited in 570 studies (between 2010 and 2012) were included; 8078 patients were treated in experimental arms. Personalized cohorts using a genomic biomarker had a higher RR, prolonged PFS and OS in comparison to protein biomarkers (all *p* < .05). Overall, patients receiving personalized targeted therapy had improved outcomes compared to non-personalized approaches or to those treated with cytotoxic agents only (both, *p* < .001). In multi-variate analyses, a personalized treatment strategy, hematologic malignancies and chemotherapy-naive patients were the only factors significantly associated with higher RR (all *p* < .001). Median PFS and OS were prolonged with a personalized approach (5.9 vs 2.7 months; p < .001 and 13.7 vs 8.9 months; p < .001, respectively). Of note, treatment related mortality was lower in personalized vs non-personalized groups (1.5% vs 2.2%; p < .001), and lower when targeted agents were compared to cytotoxic agents (1.9% vs 2.4%; *p* = .023) [32].

In 2016, the same investigators published a meta-analysis of further 13,203 patients treated in 346 early phase clinical trials. Results regarding RR, PFS and safety were comparable to the previous meta-analysis and confirmed the efficacy of a personalized treatment approach. Additionally, a higher RR was observed if treatment selection was based on genomic than on protein biomarkers [33].

In a pooled-analysis of registry data of four cancer centers, we investigated the outcome of 202 patients who were profiled by the same commercially available multiplatform technology using NGS, IHC, and FISH/CISH. In total, 82% (*n* = 166) of the patients were treated according to their MP, of which 69% (*n* = 140) were evaluable for PFS-ratio analysis. A PFS-ratio ≥ 1.3 was observed in 52% (*n* = 73) and a significant PFS improvement was achieved in the MP-guided therapy group (HR 0.70; *p* = .0024) [34].

## 7 Discussion

Most of the interventional clinical studies based on comprehensive molecular tumor profiling presented in this review showed an improvement in ORR, PFS-ratio, PFS and/or OS. Furthermore, two large meta-analyses [32,33] and a recently published pooled-analysis [34] detected a statistically significant clinical benefit for patients with advanced cancer treated with a MP-guided approach. Treatment-related mortality was lower in patients following a personalized treatment in most studies. However, the only randomized trial (SHIVA) [30] did not confirm these positive results. This study failed to show a PFS-difference in patients randomized to a MP-guided treatment approach compared to treatment at physician's choice. Many factors may have influenced this result. In a comment following the publication of the SHIVA trial, Tsimberidou and Kurzrock [35] pointed out that no rational combinations of drugs were used in the experimental arm in contrast to the control arm. It has to be kept in mind that the therapeutic efficacy of some agents strongly depends on the tumor type, the signaling network within a tumor cell and the contexture of the tumor microenvironment. Some patients of the SHIVA trial have also been incorrectly matched to a specific treatment. Other authors proposed the use of in-silico analysis algorithms [36] or that critical informations, such as the PFS-ratio as a further endpoint is lacking [37]. Thus, in the ongoing SHIVA02 - trial (NCT-03084757) the PFS-ratio is used as the primary endpoint. Data collection is estimated to be completed in April 2020. Of note, the SHIVA02 trial will not include checkpoint inhibitors as part of their treatment regimes. Recently, pembrolizumab has been approved for microsatellite instability (MSI)-high and mismatch repair-deficient tumors, and was therefore the first approved drug in the treatment of solid tumors based on predictive biomarkers regardless of tumor entity [38].

In this review, we separated the results from studies using sequencing alone as MP and those using a multiplatform profiling approach, which included tumor tissue-based methods for DNA, RNA and protein analysis. The use of multiplatform technologies seems to identify a higher number of potential targets and this subsequently might transform in a higher probability to detect an effective matching drug. However, currently there is only a limited number of targeted agents approved or tested for specific gene alterations. On the one hand, a holistic profiling approach certainly adds further knowledge to the understanding of cancer biology, but on the other hand the clinical relevance of many mutations is still unknown since the majority of detected mutations are not druggable yet. Complex bioinformatic processing of large datasets might reveal undiscovered efficacy of drugs in certain genomic constellations. However, to date there are no randomized trials available to confirm this hypothesis. Thus, the optimal profiling methodology still remains to be defined and standardized. The optimal source of tumor tissue (primary and/or metastasis) and best timing of tissue sampling also require evidence-based diagnostic guidelines. Tumor heterogeneity might be one of the major pitfalls of tumor tissue-based MP and MP-guided use of anticancer drugs. Blood-based liquid biopsy technologies using cell-free DNA or exosomes may be helpful to display tumor heterogeneity or to monitor residual disease, cancer evolution and resistance [39,40].

Quality of life in patients treated with matched agents was not regularly addressed in the studies presented. In our point of view, especially in a palliative treatment setting, the balance of efficacy, toxicity and costs of personalized cancer therapies is of great importance. Quality of life, patient-reported outcomes and total costs for patient care should be addressed in upcoming trials investigating MP-based treatment approaches. In this perspective, the meta-analyses performed by Schwaederle et al. already showed that treatment-related mortality rate and toxicity was lower when a personalized approach was used [32].

All the studies summarized here represent a current overview on individualized cancer treatment approaches for patients with malignancies. MP might become an important pillar on the way to improve drug-based cancer therapy and to broadly introduce precision medicine in oncology.

The National Cancer Institute (NCI) of the United States strongly supports precision medicine trials [41]. As an example, the “Exceptional Responders Initiative” (ERI) aims to understand the molecular processes in patients that responded to a specific drug when most other patients in a trial did not. Results might support treatment decision making and might lead to a more careful selection of patients.

The majority of studies used PFS or PFS-ratio as study endpoint. PFS-ratio strongly depends on the previous therapeutic line. Consequently, fast progression upon the last standard therapy and modest activity of the subsequent matched therapy line might lead to an acceptable PFS-ratio (i.e. >1.3). In contrast, a long lasting response on standard treatment and prolonged PFS on matched therapy might conceal efficacy of the matched therapy. Furthermore, PFS itself is strongly dependent on restaging intervals, which can differ between treatment regimens (i.e. 2 vs. 3 months). Additionally even if a statistical significant effect is observed, it remains controversial if a PFS of <3 months in highly pretreated patient collectives should be considered as a clinically relevant benefit. Overall survival is considered as the most valid efficacy parameter in oncology. However, to the best of our knowledge no randomized trial evaluated whether therapies according to MP significantly prolongs OS. Such studies are desirable and might add further evidence on beneficial effects of MP-guided treatments. In our point of view it remains challenging which endpoint parameter is the most meaningful in analyzing efficacy of matched therapy. This is also reflected by the various endpoints used in the presented studies. Using a combination of PFS (i.e. PFS lasting longer than 6 months) and PFS-ratio as a primary endpoint might be more suitable and takes into account the limitations mentioned above. Others have proposed a combination of PFS-ratio and response rates according to RECIST. In a small study the authors could conclude that combining those two endpoints might serve as surrogate for OS [42].

While the SHIVA trial investigated only a limited number of gene/protein alterations, the WINTHER-trial used, next to DNA, also RNA sequencing. In contrary the NCI-MATCH is aiming to evaluate >100 mutations in a basket study design. It remains to be seen, which study design might serve as the most appropriate when evaluating the benefit of a MP approach.

Besides the heterogeneity of study designs and outcome evaluation, algorithms for subsequent treatment decision-making and conduction of MP are not uniform. Rational algorithms and consensus guidelines are urgently needed to accomplish comparability and to increase the quality and clinical impact of MP-based clinical studies. Currently the most limiting factors in this field of clinical cancer research appear to be limited funding for investigator-initiated trials, regulatory constraints, the lack of guidelines for optimal tumor tissue sampling and the still insufficient knowledge on the development of resistance mechanisms during cancer progression [43].

In our opinion, for the near future, the use of MP and MP-guided treatments should be restricted to patients with R/R malignancies, cancers of unknown primary or orphan cancers without standard therapy options, preferably in clinical trials, as the evidence for a general use of MP in the daily routine especially for R/R cancer patients is still premature. With decreasing costs for analyzing techniques, larger biomarker panels and the development of new targeted drugs, it is tempting to assume that MP will become a standard procedure for the majority of malignancies even at early stages and that MP will decisively influence treatments with curative or palliative intent. Implementation of a molecular tumorboard within comprehensive cancer centers should further lead to improve cancer care and provides quality assurance.

## 8 Conclusion

Molecular profiling is increasingly used in different metastatic R/R tumors. The response rates, survival and quality of life benefits seem to be increased when MP-guided approaches are used. However, the only randomized trial failed to show superiority of a matched therapy approach. Hopefully, currently ongoing randomized trials will answer important open questions and add further evidence towards the significance of MP-based personalized therapy.

## Conflict of Interests

All authors declare that no conflict of interests persists

## Appendix A Supplementary Data

Targets and respective therapeutics used in different trials Image 1
