# Supplementary material for: Treatment According to Molecular Profiling in Relapsed/Refractory Cancer Patients: A Review Focusing on Latest Profiling Studies
Source: Comput Struct Biotechnol J. 2019 Mar 26;17:447–53. doi: 10.1016/j.csbj.2019.03.012 (PMC6453774; doi:10.1016/j.csbj.2019.03.012)
Supplement: Supplementary file 1 — Targets and respective therapeutics used in different trials [file mmc1.docx]

**Supplementary table**: Targets and respective therapeutics used in different trials.

| Target(s) | Agent |  |
| --- | --- | --- |
| ALK | Alectinib, Brigatinib, Ceritinib, Iorlatinib |  |
| ALK, MET, ROS1 | Crizotinib |  |
| BRCA | Talazoparib |  |
| BRAF | Dabrafenib, Encorafenib Binimetinib, Vemurafenib* |  |
| CDK4, CDK6 | Ribociclib |  |
| EGFR overexpression | Cetuximab, Necitumumab |  |
| EGFR activating mutation | Osimertinib, Erlotinib*, Gefitinib, Dacomitinib |  |
| EGFR (HER1/ERBB1), HER2 (ERBB2/neu) | Afatinib |  |
| EGFR (HER1/ERBB1), RET, VEGFR2 | Vandetanib |  |
| FLT3, KIT, MET, RET, VEGFR2 | Cabozantinib |  |
| HER2 (ERBB2/neu) | Ado-trastuzumab emtansine, Neratinib, Pertuzumab, Trastuzumab* |  |
| HER2 (ERBB2/neu), EGFR (HER1/ERBB1) | Lapatinib* |  |
| KIT, PDGFR, ABL | Imatinib* |  |
| KIT, PDGFRβ, RAF, RET, VEGFR1/2/3 | Regorafenib |  |
| KIT, PDGFRβ, VEGFR1/2/3 | Axitinib |  |
| MEK | Cobimetinib, Trametinib |  |
| mTOR (also PTEN, STK11, RICTOR, RAPTOR, PI3KCA, AKT1, INPP4B) | Everolimus* |  |
| mTOR | Temsirolimus |  |
| NTRK | Larotrectinib |  |
| PARP | Niraparib, Olaparib, Rucaparib |  |
| PDGFRα | Olaratumab |  |
| PDGFRα, FLT3 | Sorafenib* |  |
| PIGF, VEGFA/B | Ziv-aflibercept |  |
| PTCH, smoothened receptor | Vismodegib |  |
| SLAMF7 (CS1/CD319/CRACC) | Elotuzumab |  |
| SRC (also ABL) | Dasatinib* |  |
| VEGF ligand | Bevacizumab |  |
| VEGFR, PDGFR, KIT | Pazopanib |  |
| VEGFR, PDGFR, KIT, RAF | Sorafenib |  |
| VEGFR2 | Lenvatinib |  |

* Targeted agents used in the SHIVA-trial

Abbreviations: ALK: Anaplastic-lymphoma kinase, Met: Tyrosine-protein kinase, BRCA: BReast CAncer, CDK: Cycline dependent kinase, EGFR: Epidermal growth factor receptor, HER2: Human epidermal receptor 2, RET: Rearranged during transfection, VEGFR: Vascular endothelial growth factor receptor, PDGFR: Platelet derived growth factor receptor; ABL: Abelson murine leukemia viral oncogene homolog, mTOR: mammalian target of rapamycin, PTEN: Phosphatase and tensin homologue, STK11: Serine threonine kinase 11, RICTOR: Rapamycin-insensitive companion of mammalian target of rapamycin, RAPTOR: Regulatory-associated protein of mTOR, PI3KCA: Phosphatidylinositol-4,5-bisphosphate 3-kinase catalytic subunit alpha, INPP4B: Inositol polyphosphate-4-phosphatase type II B, NTRK: Neurotrophic tyrosine kinase, PARP: Poly(ADP-ribose)-polymerase 1, FLT3: Fms related tyrosine kinase 3, PlGF: Placental growth factor, PTCH: Human homologue of the drosophila segment polarity gene patched, SLAMF7: Signaling lymphocytic activation molecule-F, SRC: Sarcoma-kinase.
